# Rational Design of a Fibroblast Growth Factor 21-Based Clinical Candidate, LY2405319

**DOI:** 10.1371/journal.pone.0058575

**Published:** 2013-03-11

**Authors:** Alexei Kharitonenkov, John M. Beals, Radmila Micanovic, Beth A. Strifler, Radhakrishnan Rathnachalam, Victor J. Wroblewski, Shun Li, Anja Koester, Amy M. Ford, Tamer Coskun, James D. Dunbar, Christine C. Cheng, Christopher C. Frye, Thomas F. Bumol, David E. Moller

**Affiliations:** Lilly Research Laboratories, Lilly Corporate Center, Indianapolis, Indiana, United States of America; University of Hong Kong, China

## Abstract

Fibroblast growth factor 21 is a novel hormonal regulator with the potential to treat a broad variety of metabolic abnormalities, such as type 2 diabetes, obesity, hepatic steatosis, and cardiovascular disease. Human recombinant wild type FGF21 (FGF21) has been shown to ameliorate metabolic disorders in rodents and non-human primates. However, development of FGF21 as a drug is challenging and requires re-engineering of its amino acid sequence to improve protein expression and formulation stability. Here we report the design and characterization of a novel FGF21 variant, LY2405319. To enable the development of a potential drug product with a once-daily dosing profile, in a preserved, multi-use formulation, an additional disulfide bond was introduced in FGF21 through Leu118Cys and Ala134Cys mutations. FGF21 was further optimized by deleting the four N-terminal amino acids, His-Pro-Ile-Pro (HPIP), which was subject to proteolytic cleavage. In addition, to eliminate an *O*-linked glycosylation site in yeast a Ser167Ala mutation was introduced, thus allowing large-scale, homogenous protein production in *Pichia pastoris*. Altogether re-engineering of FGF21 led to significant improvements in its biopharmaceutical properties. The impact of these changes was assessed in a panel of in vitro and in vivo assays, which confirmed that biological properties of LY2405319 were essentially identical to FGF21. Specifically, subcutaneous administration of LY2405319 in *ob/ob* and diet-induced obese (DIO) mice over 7–14 days resulted in a 25–50% lowering of plasma glucose coupled with a 10–30% reduction in body weight. Thus, LY2405319 exhibited all the biopharmaceutical and biological properties required for initiation of a clinical program designed to test the hypothesis that administration of exogenous FGF21 would result in effects on disease-related metabolic parameters in humans.

## Introduction

Since the initial identification of FGF21 as a novel metabolic regulator [1], this protein has become the focus of intense research in the area of glucose and lipid homeostasis and an important target for drug discovery initiatives [2]. Indeed, the potential metabolic benefits of FGF21 pharmacology in preclinical models are striking and provide a compelling rationale for ongoing life sciences research. When administered to rodents and non-human primates, exogenous FGF21 induces potent lowering of blood glucose, triggers improvements in insulin sensitivity, and enhances pancreatic β-cell function and mass. Additional pharmacologic effects of FGF21 include a reversal of hepatosteatosis and a reduction in adipose tissue mass. Importantly, triglyceride lowering and a beneficial shift in plasma lipoprotein profiles suggest the potential of an FGF21-based therapy for amelioration of cardiovascular disease risk [2].

The initial evaluation of formulation properties indicated a low probability for development of an FGF21 molecule suitable for clinical use. Furthermore, as the Type 2 diabetic patient population is exponentially growing [3], there was a need to select a cost-effective scalable expression platform and purification manufacturing process for an FGF21-based protein. Thus, the strategic intent of our FGF21 biopharmaceutical re-engineering efforts were driven by the desire to create a stable, concentrated, and preserved solution preparation, while maintaining a favorable bioactivity profile that was amenable to a wide range of dosing demands in future clinical trials and potentially broader populations of patients.

Here we describe the design, structural characteristics, biopharmaceutical and pharmacologic properties of a novel FGF21 variant, LY2405319. The biopharmaceutical optimization of FGF21 was aimed at enhancing physical stability of the protein, enabling its expression in yeast (*Pichia pastoris*) all while maintaining its *in vivo* biological potency relative to the native molecule yielding our engineered FGF21 analog, LY2405319. LY2405319 was selected for further testing in early stage clinical trials. The results of these clinical development efforts will be reported in future publications with the intent of shedding light on the therapeutic utility of FGF21-based therapies.

## Materials and Methods

### Homology Modeling

LY2405319 structure was generated from a homology model (residues 18–140) based on known FGF structures using a proprietary algorithm.

### Biophysical Screen

Biophysical screening focused on evaluation of physical stability in the presence of preservative (m-cresol) under physiological subcutaneous conditions (37°C; 150 mM NaCl). Dynamic light scattering, at 90°C, was used to monitor aggregation at 10 mg/ml for up to 24 hours at 37°C in PBS with m-cresol.

### Differential Scanning Calorimetry

The DSC thermograms of FGF21 and LY2405319 were collected on a VP-DSC Instrument (Microcal, Piscataway, NJ). The concentration of protein was between 1–2 mg/mL in PBS buffer. Both PBS and sample solutions were degassed before loading in the cells. The scanning parameters for the DSC experiment were as follows: the temperature scan range was 10–95°C, scan rate was 60°C/hr, pre-scan thermostat was 15 minutes, post-scan thermostat was 10 minutes, and the filtering period was set at 16 sec. The experimental parameter included number scan of 12–20 and a post-cycle thermostat of 10°C. The cells were filled with temperatures between 10–20°C.

### Protein Expression in E. coli & Purification

FGF21 was purified from a BL21 *E. coli* expression system from granules isolated from cells, which were collected by centrifugation. Granules were solubilized and sulfitolyzed in TRIS/7 M Urea at pH 9. All chromatography steps were done at room temperature. The solubilized protein was initially purified by Fast Flow Q Sepharose prior to refolding. FGF21 was diluted to approximately 0.5 mg/mL with 20 mM glycine at pH 9 and then folded by addition of cysteine to a final concentration of 2 mM. The refolding solution was incubated at 4°C for 48 hours with 1 buffer exchange. The folded protein was purified using preparative reversed-phase chromatography on a Vydac C18 column. pH of the reversed-phase pool was adjusted to greater than pH 7. The pooled FGF21 fraction was subsequently purified using Superdex 75 size exclusion chromatography. Mono Q anion exchange chromatography was used for the final polishing step. The final pool was dialyzed overnight at 4°C into PBS, pH 7.4. For FGF21 variants expressed in the HEK293/EBNA expression system, the same chromatography steps were followed as described above excluding the protein fold. The average purity (by HPLC) of FGF21 proteins was >95%.

### Engineering for Expression in Pichia Pastoris

All molecular biology work was carried out utilizing standard methods, including DNA cloning, PCR, protein expression evaluations, and analyses. Modifications to the native FGF21 gene (i.e., construction of variants) were made using standard PCR-based methodologies (overlap extension mutagenesis) and genes were designed to be cloned directly into *Pichia* expression plasmid backbones. Alternatively genes were constructed synthetically (GeneArt, Regensburg, Germany).

Recombinant strains were screened for FGF21 variant expression utilizing a variety of methods that included silver and Coomassie-stained polyacrylamide gels, Western blot analyses, and ELISA [4], or HPLC analyses. A glucose uptake assay was used to determine the activity of native FGF21 and all variants produced.

A *Pichia* expression system (Invitrogen, Carlsbad, CA) was used for protein production. The parental expression plasmids utilized the α-mating factor prepro-peptide as the secretion signal and utilized the GAPDH promoter for the constitutive expression of the gene of interest [5]. In addition, these expression plasmids contained a second expression cassette for the production of green fluorescent protein (GFP) under the control of the AOX1 promoter. GFP co-expression was used as a screening tool in identifying high-producing recombinant strains. Regulation of the AOX1 promoter by methanol addition allows GFP to be expressed during the strain screening process, but not expressed during the manufacturing process. The expression host utilized was SMD1163, a derivative of the GS115 strain containing additional mutations that inactivate the Pep4 and Prb1 vacuolar proteases, which literature have suggested can impact protein quality [6].

Fermentations were inoculated from shake-flasks, following a two-stage vegetative culture growth. After inoculation, the cells go through an exponential growth consuming a pre-defined feed of glucose. When the glucose was consumed, a dissolved oxygen (DO) spike was observed followed by a pH spike, indicating the consumption of organic acids produced during growth on glucose. The pH spike signals the controller to decrease the temperature from 30°C to 22°C, to ramp up airflow and agitation and to supply a limiting glucose feed. The pH was maintained at 7.0 during most of the process using ammonium hydroxide addition. Cell mass and FGF21 variant titers increased throughout the fermentation. In general, the run proceeds for 48 hours after the pH spike (approximately, 75 hours in total).* .*

### In vitro Biology

#### Cell lines and proteins

3T3-L1 fibroblasts and HepG2 hepatoma cells were from ATCC (Manassas, VA). 3T3-L1 adipocytes were differentiated as previously described [7]. 3T3-L1/βKlotho fibroblasts were generated as previously described [8] and 3T3-L1/Klotho fibroblasts were generated with the same method. FGF21 was generated as previously described [1]. Insulin was from Sigma (St Louis, MO); FGF1, FGF2 and FGF23 were from R&D Systems (Minneapolis, MN).

#### ERK1/2 phosphorylation assay

Cells were treated as indicated for 5 min and subsequently lysed. Total ERK phosphorylation was assessed using an AlphaScreen SureFire Phospho-ERK1/2 Assay Kit (PerkinElmer, Waltham, MA) according to the manufacturer’s instructions and an EnVision Multilabel Microplate Reader Model 2103 (Perkin Elmer) with the AlphaScreen HTS Turbo option was used for signal detection.

#### RNA isolation, RT and real-time quantitative PCR

RNA was isolated from cells using TRIzol reagent (Invitrogen, Carlsbad, CA) and was reverse transcribed into cDNA using a High-Capacity cDNA Reverse Transcription Kit (PE Applied Biosystems, Foster City, CA). Reactions were performed in triplicate on an ABI Prism 7900HT (PE Applied Biosystems) and were normalized to either 36B4 mRNA or 18S rRNA. Assays-on-Demand Gene Expression Products (PE Applied Biosystems) were as follows: GLUT1 (Hs00197884_m1).

#### Mitogenicity assay

Cells were treated as indicated for 16 h, followed by incubation with 1 uCi per well [methyl-^3^H] thymidine (MP Biomedicals, Solon, OH) for 4 h, before being lysed. DNA was harvested and ^3^H incorporation was measured using a Wallac 1450 Microbeta Scintillation Counter (Perkin Elmer).

### In vivo Biology

All experimental animal protocols in this study were approved by Eli Lilly and Co. Animal Use and Care Committee. 18-week old diet-induced obese (DIO) male C57/BL6 mice (Taconic, NY) were maintained on a high fat diet (TD95217; Harlan Teklad, Madison, WI) from weaning and had free access to food and water for at least 12 weeks before randomization by weight. Male *ob/ob* mice (Harlan) were maintained on Teklad 2014 chow with free access to food and water for a minimum of 5 weeks before randomization by blood glucose. Animals were individually housed in a temperature-controlled (24°C) facility with a 12∶12 light:dark cycle. DIO and *ob/ob* mice were treated, for 14 or 7 days, respectively, with vehicle or various doses of FGF21 and FGF21 variants, as indicated, via continuous subcutaneous infusion with mini-osmotic pumps (Alzet, Cupertino, CA). Food intake and body weight were recorded daily. Body composition of DIO mice was determined using Quantitative Nuclear Magnetic Resonance analysis (ECHO MRI, 3-1 Composition Analyzer; Echo Medical Systems, Houston, TX) on the initiation of treatment (Day 1) and on the last day of treatment (Day 14). Blood glucose was monitored daily using Precision G Blood Glucose Testing System (Abbott Laboratories, Abbott Park, IL). Insulin levels were measured by ELISA (Crystal Chem Inc., Downers Grove, IL) from plasma samples collected on the final day of treatment. Plasma FGF21 and LY2405316 levels were determined by ELISA using internally generated antibodies [4].

### Statistical Analysis

Data are presented as mean ± SEM. Statistical analysis was performed using one-way ANOVA, followed by Dunnett’s or Tukey’s multiple comparison test or two-way ANOVA followed by Bonferonni multiple comparison test. Significant differences of P<0.05 are identified with an asterisk.

## Results

### Biophysical Stability of FGF21 Under Physiological Conditions

Adequate and effective treatment of the Type 2 diabetes (T2D) patient population with a protein therapy amenable to once-daily (QD) administration requires the development of a multi-use preserved formulation. However, it is well established that phenolic preservatives in pharmaceutical preparations can induce the formation of protein-based insoluble aggregates [9]–[13] that can lead to undesired immunological reactions [14]; consequently, a successful recombinant protein engineered for broader clinical use should exhibit a robust stability in the formulation and under subcutaneous conditions.

**Figure 1:**
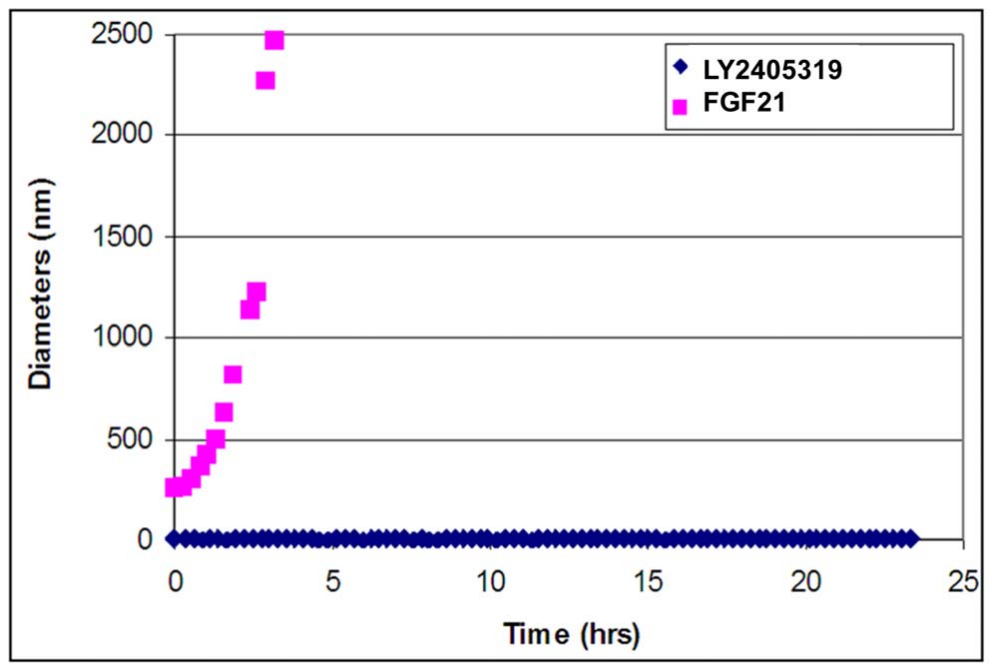
Dynamic light scattering assessment of FGF21 and LY2405319. Formulation stability of FGF21 (squares) and LY2405319 (diamonds) was assessed via dynamic light scattering at 37°C at 10 mg/mL in phosphate buffered saline at pH 7.4 containing 30 mM m-cresol. Protein concentration was 10 mg/ml.

To assess the physical stability characteristics of FGF21 in a solution containing preservative, the protein was formulated at 10 mg/mL in phosphate buffered saline (PBS) containing 30 mM m-cresol at pH 7.4. Physical stability was assessed by dynamic light-scattering at 37°C. Our data suggest that FGF21 rapidly aggregates under these conditions (Figure 1). These results suggest that the preservative may be unfavorably altering the conformational state of FGF21 under conditions analogous to exogenous administration thus promoting undesired protein-protein interactions.

### Thermal Stability of FGF21

**Figure 2:**
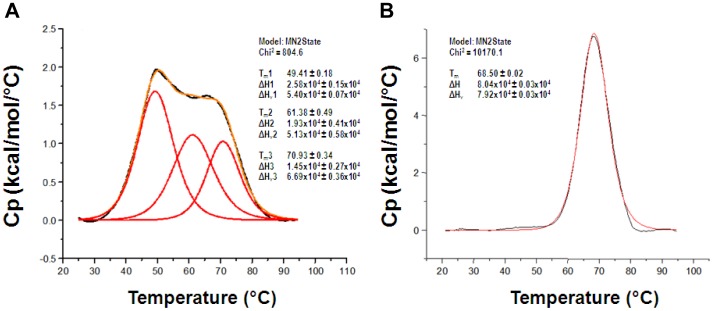
Thermal stability profile of FGF21 and LY2405319. Differential Scanning Calorimetry (DSC) was used to assess thermal stability of FGF21 (A) and LY2405319 (B); protein concentration was 1.3–1.6 mg/ml in PBS. Scan rate was 60°C/h.

To assess the conformational stability of FGF21, the protein was subjected to differential scanning calorimetry (Figure 2A). The results show that the protein undergoes at least three thermal-unfolding states suggesting a conformationally dynamic protein. More notably, FGF21 unfolding is apparent at 37°C, potentially due to access of the hydrophobic phenolic preservative to the hydrophobic core of the protein and, subsequently, destabilization of its native structure leading to aggregation [12]. The access of a small phenolic molecule to the hydrophobic core of proteins has been well documented with T4 lysozyme [15].

### Engineering Conformational Stability

The initial evaluation of FGF21 established that the successful generation of its preserved commercial formulation would be unlikely due to protein aggregation in the presence of preservative under physiological conditions (Figure 1). This was further exacerbated by the poor conformational/thermal stability of FGF21 (Figure 2A). To improve the probability of technical success of creating a preserved formulation of an FGF21 variant, we optimized FGF21 aiming to increase its conformational stability in the presence of preservatives and under concentrated, physiologic conditions, while maintaining a favorable bioactivity profile.

**Figure 3:**
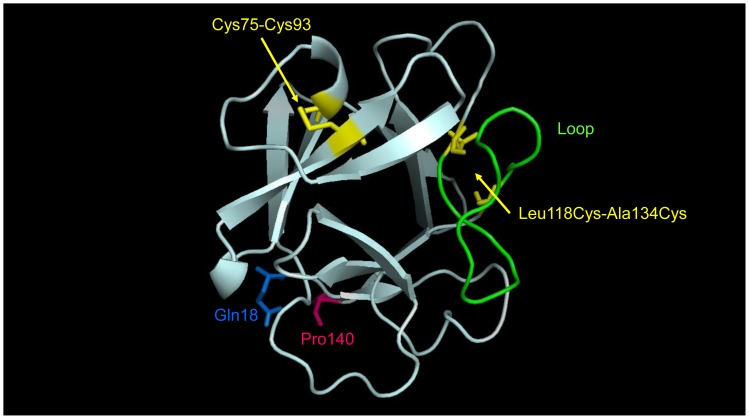
FGF21 homology model. An engineered disulfide bond in LY2405319 achieved loop stabilization at the C-terminus. The FGF21 homology model includes residues 18–140 of the human sequence. The native disulfide at C75-C93 and the new engineered disulfide bond at Leu118Cys-Ala134Cys are highlighted in yellow. The intervening loop between Leu118Cys and Ala134Cys is highlighted in green.

To improve conformational stability, and subsequent biopharmaceutical properties, a disulfide stabilization engineering strategy was used. This approach utilized the FGF21 homology model (Figure 3) derived from existing X-ray structures of various FGF molecules and sequence alignments between human, mouse and rat FGF21 orthologs [16]. Based on distance and orientation constraints assessed via the structure modeling, we modified the FGF21 sequence via point-specific mutagenesis to introduce an additional disulfide bond. This yielded a variant where the engineered disulfide bond at Leu118Cys-Ala134Cys stabilized a loop at the C-terminal domain of FGF21.

### Engineering for Expression in Pichia Pastoris

Initial attempts to express FGF21 in *Pichia pastoris* resulted in low productivity (<1 mg/L at shake-flask scale) in addition to numerous issues related to the integrity/heterogeneity of the secreted product. Specifically, in yeast, FGF21 underwent significant proteolysis, with the major clipping observed at the N-terminus of the protein resulting in almost complete removal of the first four amino acids, HPIP. A second major quality issue was the high level of *O*-linked glycosylation observed (>70%).

**Figure 4:**
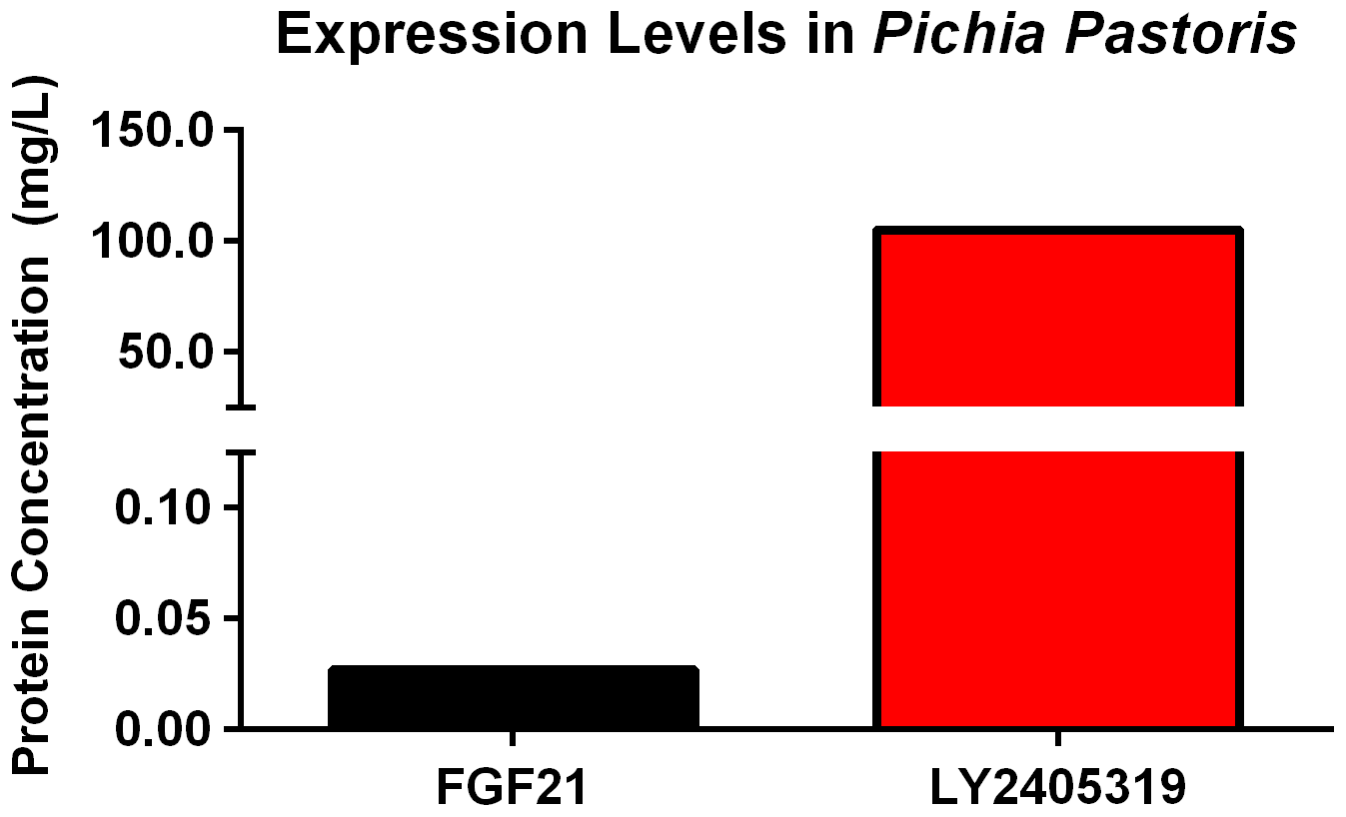
Expression levels of FGF21 and LY2405319 in *Pichia pastoris.* FGF21 (black) and LY2405319 (red) were expressed in *Pichia pastoris* at shake-flask scale. Protein amounts were quantitated from crude media using an FGF21 ELISA.

The introduction of a second disulfide bond in FGF21 resulted in major improvements in productivity and protein quality in *Pichia pastoris*. Productivity was increased by more than three orders of magnitude (>100 mg/L at shake-flask scale; Figure 4). This rise correlated with improved thermal stability of the protein [17]. However, despite higher yields compared to native FGF21, the quality of the FGF21 variant produced in *Pichia pastoris* remained inadequate due to high levels of N-terminal proteolysis and *O*-linked glycosylation.

To address protein quality challenges, the FGF21 variant was further re-engineered to decrease the level of *O*-linked glycosylation through specific amino acid substitutions at key positions. Serine at position 167 (Ser167) was identified via peptide mapping as the major *O*-linked glycosylation site and was subjected to site-specific mutagenesis. Out of 11 constructs with single amino acid substitution that were designed and screened, the preferred variant, Ser167Ala, was chosen based on neutrality of amino change and impact on protein activity. Protein characterization of the FGF21 variant produced in *Pichia pastoris* displayed low levels of *O*-linked glycosylation (<3%), confirming Ser167 as the primary glycosylation site in yeast.

To address the heterogeneity of secreted product associated with N-terminal proteolysis due to aminopeptidase activity, FGF21 was further modified to remove the first four N-terminal amino acids, ΔHPIP. The decision to include this truncation was based on the knowledge that ΔHPIP FGF21 is fully biologically active [18], [19].

Subsequently, the optimized FGF21 variant for *Pichia pastoris* expression, FGF21 Leu118Cys-Ala134Cys, Ser167Ala, ΔHPIP, or LY2405319, was produced at manufacturing-scale in fermentation tanks (at greater than 1 g/L) and was essentially devoid of the *O*-glycosylation/N-terminal processing heterogeneity issues associated with native FGF21.

### Biophysical Characteristics of LY2405319

To assess the impact of the modifications on biophysical properties, yeast-produced LY2405319 was evaluated by dynamic light-scattering at 37°C (Figure 1). The results demonstrate enhanced physical stability of LY2405319 compared to FGF21, which rapidly aggregated under these conditions. Our data also suggest that the engineered disulfide bond in the C-terminal domain of FGF21 stabilizes the protein from aggregation induced by phenolic preservative. Moreover, the engineered disulfide bond clearly improves the conformational instability of FGF21 as exemplified by the collapse of the thermal unfolding from a three-state, conformationally dynamic profile into a single conformation state for LY2405319 with a Tm of 68.5°C, and little to no thermal unfolding at 37°C (Figure 2B). These results suggest that the engineered disulfide significantly improves the conformational stability of LY2405319 and likely minimizes phenolic preservative-induced aggregation by limiting its access to the hydrophobic core of the FGF21 variant. Thus, intelligently re-engineering FGF21 to LY2405319 yields a homogenous protein product that is amenable to yeast production and possesses the necessary stability required for the multi-use formulation of a therapeutic protein.

### Comparing Bioactivities of FGF21 and LY2405319

As the goal of FGF21 engineering was to improve biopharmaceutical properties of the protein while maintaining its *in vivo* potency, LY2405319 was profiled for bioactivity in cell culture studies and in mouse models of diabetes in a panel of *in vitro* and *in vivo* assays that have been previously established to characterize the effects of FGF21 [1], [18]–[21].

### In vitro

**Figure 5:**
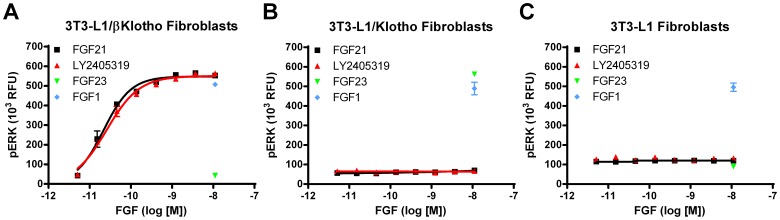
LY2405319 bioactivity is βKlotho dependent. Klb-dependence was evaluated by treating 3T3-L1/Klb (A), 3T3-L1/Kl (B), and 3T3-L1 (C) fibroblasts for 5 min with various concentrations of FGF21 (black squares) and LY2405319 (red triangles). Cells were also treated for 5 min with 10 nM FGF1 (blue diamonds) to assess klotho cofactor-independent activity and 10 nM FGF23 (green inverted triangles) to assess Kl-specific activity. After treatment, cells were lysed, and ERK1/2 phosphorylation was measured using AlphaScreen technology. Values are presented as mean ± SEM in relative fluorescent units.

LY2405319 was evaluated head-to-head with FGF21 in an ERK1/2 phosphorylation assay in 3T3-L1 cells with stable over-expression of βKlotho (Klb), a critical co-factor for FGF21 action [8], [22]–[24]. Both, LY2405319 and FGF21 stimulated ERK1/2 phosphorylation in 3T3-L1 cells expressing Klb (Figure 5A). In this assay, LY2405319 and FGF21 demonstrated equivalent potency (Table 1).

**Table 1:** Summary of *in vitro* activities of FGF21 and LY2405319.

		FGF21			LY2405319			
Activity	Cell Line	EC_50_ (nM)	Lower CI (nM)	Upper CI (nM)	EC_50_ (nM)	Lower CI (nM)	Upper CI (nM)	*P*
ERK1/2 Phospho	3T3-L1/Klb	0.021	0.016	0.029	0.026	0.019	0.035	0.480
Glucose Uptake	3T3-L1/Klb	1.094	0.131	9.179	1.912	0.482	7.591	0.425
Glucose Uptake	3T3-L1 Adipo	0.621	0.447	0.862	0.864	0.424	1.745	0.554
GLUT1 Transcript	HepG2	2.465	1.205	5.040	1.819	1.220	2.711	0.743

CI: 95% confidence interval;

* *P*≤0.05.

LY2405319 and FGF21 were both inactive on either cells expressing αKlotho (Kl), a related family member necessary for FGF23 but not FGF21 action (Figure 5B) [25], or on parental L1 fibroblasts that are devoid of Klb expression (Figure 5C) [8]. Importantly, these cells responded to stimulation with FGF1 by ERK1/2 phosphorylation (Figure 5A–C), a canonical non-Kl/Klb-dependent member of the FGF superfamily [26], confirming the presence of functional FGFRs in all three cell lines utilized in these experiments. Finally, only 3T3-L1/Kl (Figure 5B) but not parental 3T3-L1 or 3T3-L1/Klb fibroblasts (Figure 5A & C) were activated by FGF23 corroborating the presence of a functional and FGF23-specific Klotho co-factor in 3T3-L1/Kl cells. Taken together, these observations are consistent with the specific requirement of Klb, and not of Kl, for both FGF21 and LY2405319 activity.

**Figure 6:**
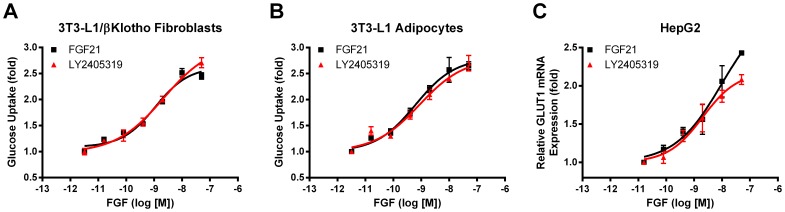
LY2405319 bioactivity is comparable to wild type FGF21. LY2405319 and FGF21 bioactivities were compared in mouse 3T3-L1/Klb fibroblasts (A) and differentiated mouse 3T3-L1 adipocytes (B) by monitoring 2-Deoxy-D-[U-^14^C]-glucose accumulation and in human HepG2 hepatoma cells by examining relative expression of GLUT1 mRNA (C) after incubation for 3 h or 1 h, respectively, with various concentrations of FGF21 (black squares) or LY2405319 (red triangles). Mean values ± SEM are presented as fold relative to treatment with vehicle alone.

Next, LY2405319 was evaluated for its ability to stimulate glucose uptake in cells expressing Klb, as has been previously demonstrated with FGF21 [18]. LY2405319 stimulated glucose uptake in 3T3-L1/Klb fibroblasts with equivalent potency to FGF21 (Figure 6A; Table 1). The differentiation of 3T3-L1 fibroblasts to adipocytes results in increased expression of endogenous Klb thus conferring FGF21 responsiveness [8]. Therefore, LY2405319-induced glucose uptake was next examined in differentiated mouse 3T3-L1 adipocytes, a cell line in which the bioactivity of FGF21 was initially found via a potency screen [1]. LY2405319 in this system also demonstrated a comparable potency to FGF21 (Figure 6B; Table 1).

Having shown bioactivity equivalent to native FGF21 in mouse 3T3-L1 cells, LY2405319 action was next evaluated in HepG2 hepatoma cells that are of human origin and endogenously express Klb and FGFRs [27], [28]. Again, LY2405319 demonstrated equivalent potency to FGF21 as measured in a GLUT1 transcription assay, a known readout of FGF21 activity [1] (Figure 6C; Table 1).

**Figure 7:**
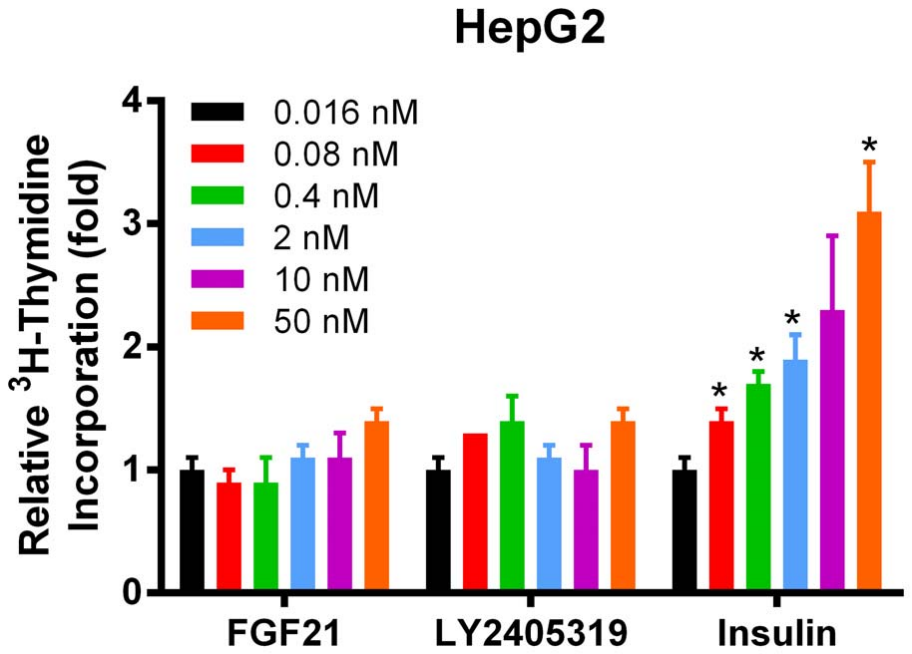
LY2405319 is not mitogenic in human HepG2 hepatoma cells. HepG2 human hepatoma cells were treated with 0.4nM FGF2 or various concentrations of FGF21, LY2405319, or insulin for 16 h and pulsed with [methyl-^3^H] thymidine for 4 h prior to DNA collection and measurement of ^3^H incorporation.

Finally, LY2405319 was examined for possible mitogenic activity in HepG2 cells. Under conditions where 0.4 nM FGF2 and 50 nM insulin induced a rise in [methyl-^3^H] thymidine incorporation (2.4 and 3 fold, respectively), no significant increase was observed when HepG2 cells were treated with LY2405319 or FGF21 up to a maximal dose of 50 nM (Figure 7).

Collectively, these data indicate that LY2405319 and FGF21 have comparable *in vitro* potency in both mouse and human cells, and that Klb-dependence together with the non-mitogenic character of FGF21 action remained intact in LY2405319 following protein engineering.

### In vivo

Since FGF21 is known to induce metabolic actions in *ob/ob* and diet induce obese C57BL6 (DIO) mice [1], [20], [21], [24], [29], LY2405319 was next tested for pharmacological effects in both animal strains.

**Figure 8:**
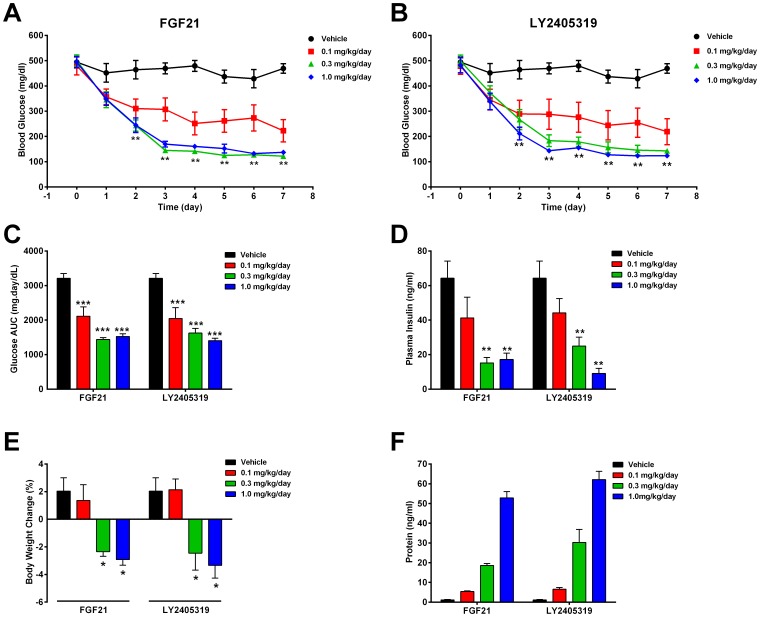
Treatment of *ob/ob* mice with either FGF21 or LY2405319 improves metabolic dysfunction. Male *ob/ob* mice were treated with FGF21 or LY2405319 at various concentrations, as indicated, by constant infusion for 7 days. Blood glucose is reported as daily levels (A, B) or cumulatively for the treatment period (C). Plasma insulin was measured on the final day of treatment (D). Body weights were measured daily and are presented as body weight change (E) over the course of treatment. Plasma FGF21 and LY2405319 levels on the final day of treatment were measured by ELISA (F). Values are reported as mean ± SEM. (*), (**), and (***), p<0.01, p<0.001, and p<0.001, respectively, as compared to vehicle.

When dosed chronically to *ob/ob* mice for 7 days via continuous subcutaneous infusion, FGF21 and LY2405319 treatment resulted in an effective lowering of glycemia that amounted to a normalization of non-fasted glucose levels at the two highest doses (Figure 8A & B). Comparative analysis of day 0 to 7 AUC (area under the curve) of daily non-fasted glucose values revealed that the efficacy profiles of FGF21 and LY2405319 at each comparable dose were not significantly different (Figure 8C). Lowering of plasma insulin was indistinguishable in animal cohorts that were administered equivalent doses of FGF21 and LY2405319 (Figure 8D). In addition to glucose and insulin lowering, *ob/ob* mice infused with 0.3 or 1 mg/kg doses of FGF21 or LY2405319 exhibited similar body weight loss of approximately 3–5% (Figure 8E). Finally, levels of human FGF21 and LY2405319 in mouse plasma were comparable at each dose and proportional to the dose administered (Figure 8F) indicative that FGF21 and its engineered variant have identical pharmacokinetic properties.

**Figure 9:**
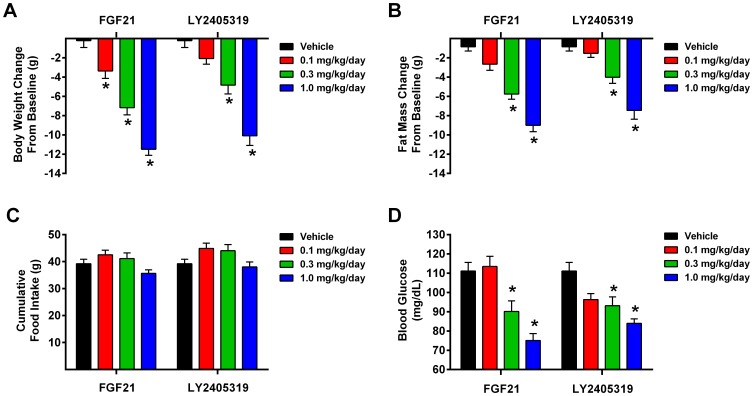
Treatment of DIO mice with either FGF21 or LY2405319 improves metabolic dysfunction. Male DIO mice were treated with FGF21 or LY2405319 at various concentrations, as indicated, by constant infusion for 14 days. Changes in body weight (A), fat mass (B) cumulative food intake (C), and blood glucose (D) were determined for the duration of treatment. Values are reported as mean ± SEM. (*), p<0.01 as compared to vehicle.

In DIO mice, FGF21 and LY2405319 led to a profound time- and dose-dependent body weight reduction over two weeks of administration. The degree of weight loss achieved was not statistically different when comparing the same dose levels of each molecule. At the highest dose, 1 mg/kg, net weight loss amounted to approximately 30% of initial body mass (Figure 9A). This effect was primarily a consequence of reduced adiposity (Figure 9B). Neither FGF21 nor LY2405319 affected total food intake (Figure 9C), as we have reported previously for FGF21 [20], [24]. In fact, caloric consumption was elevated when normalized to body weight changes, a finding also consistent with earlier results [20]. Finally, LY2405319 treatment led to significant plasma glucose lowering in the non-fasted state at 0.3 and 1 mg/kg/day doses (Figure 9D). The net effects of LY2405319 and FGF21 on plasma glucose in DIO mice were comparable but relatively minor (about 30 mg/dL), even at the highest 1 mg/kg/day dose, compared to more pronounced glucose lowering effects in *ob/ob* mice (Figure 8A & B). This difference is likely a reflection of the inability of FGF21 to induce hypoglycemia in animals [1]. Thus, the relative window for an anti-glycemic effect in DIO animals is smaller compared to *ob/ob* mice which present with a truly diabetic phenotype. Regarding body weight effects, *ob/ob* mice were more obese at baseline when compared to DIO mice; however, FGF21 and LY2405319 produced greater weight loss effects in DIO mice (Figure 9A) likely due to the fact that FGF21 is known to be ineffective in promoting energy expenditure in leptin-deficient animals [30].

As measured by FGF21 ELISA at the end of the study in DIO mice, the steady-state levels of plasma FGF21 and LY2405319 were proportional to the administered dose and comparable (data not shown) similar to the findings in the ob/ob mouse experiment (Figure 8F). Thus, the results of LY2405319 administration in two strains of mice are consistent with known FGF21 pharmacology [20], [21], and the efficacy of LY2405319 was comparable to that of FGF21.

## Discussion

The goals of our engineering efforts were to identify a novel FGF21 variant with improved pharmaceutical properties that simultaneously maintains the selectivity, potency, and pharmacology of FGF21 in the optimized molecule. Initially, we focused our research on native FGF21 in order to carefully characterize its physico-chemical properties and expression characteristics which are important for development of a commercial product.

Characterization of FGF21 in biophysical screens showed that the protein was prone to formation of insoluble protein aggregates under physiologic conditions, particularly in a preserved formulation format, *i.e.,* in the presence of phenolic preservative required for repeat dose administration of a daily parenteral product (Figure 1). In addition, we demonstrated that the thermal stability profile of FGF21 is suboptimal with evidence of unfolding observed under physiological conditions (Figure 2A). In parallel to improving the biophysical properties of FGF21, the molecule was optimized for protein expression in yeast. The use of microbial expression systems to support the manufacture of recombinant protein therapeutics is well established and these systems, primarily *Escherichia coli* and yeast, are leveraged to manufacture products such as human insulin and growth hormone [31]. However, *E. coli* manufacturing processes are typically based on the production of insoluble protein that requires denaturation and refolding to achieve an active form. In contrast, *Pichia pastoris* can be used to secrete properly folded and fully active recombinant proteins into the culture medium thus making them amenable to the types of manufacturing processes and facilities used to produce mammalian-derived recombinant proteins such as monoclonal antibodies. Such an expression system provides greater flexibility and offers the potential for shorter expression periods, lower manufacturing costs, and a more scalable expression process compared to mammalian cell-based processes [32]. Therefore, we elected to leverage the *Pichia* system for use in developing an expression platform for an FGF21-based therapeutic protein. Nevertheless, despite all the potential advantages of *Pichia pastoris*, our attempts to express native FGF21 in this system were compromised by poor yield and heterogeneity of the protein product brought about by cleavage of the molecule in addition to *O*-linked glycosylation.

In order to overcome the pharmaceutical limitations that were evident with FGF21, a number of critical changes in the molecule were designed and subsequently engineered. These efforts culminated in the identification of LY2405319. The changes featured in this analog include the addition of a new disulfide bond (Leu118Cys-Ala134Cys) (Figure 3), deletion of the four N-terminal amino acids, His-Pro-Ile-Pro, and an additional Ser167Ala point mutation. Collectively, the engineering changes featured in LY2405319 were associated with marked improvements in its physical and conformational stability (Figure 2B), increased compatibility with the preservative in m-cresol-containing formulations (Figure 1), eliminated N-terminal proteolysis and considerably reduced yeast-based *O*-link glycosylation. These changes also ensured greater yields of homogeneous protein material upon LY2405319 production in the *Pichia pastoris* expression system (Figure 4).

Having identified LY2405319 as a novel biopharmaceutically improved variant of FGF21, we next systematically profiled its biologic activity in a series of *in vitro* and *in vivo* assays that had been previously developed to characterize the biologic activity of the native protein. Overall, LY2405319 appeared to produce an identical degree of potency and selectivity compared to FGF21 in cell-based assays which measured Klb-dependent activation of FGF receptors. The activity of LY2405319 was also confirmed in an adipocyte model of glucose uptake (Figure 6A & B) along with its potency in liver derived cultured cells (Figure 6C), which express endogenous FGF receptors and Klb. Importantly, we also examined the mitogenic potential of LY2405319 and FGF21 in HepG2 cells; however, both proteins at pharmacologic doses showed no stimulation of proliferation (Figure 7). Both LY2405319 and FGF21 were subsequently shown to exert nearly identical pharmacologic effects when administered to two distinct mouse models of obesity and Type 2 diabetes. Thus, we were able to demonstrate that administration of LY2405319 produced substantial effects *in viv*o to ameliorate hyperglycemia and to reduce excess adiposity, similar to FGF21. The time course, potency, and extent of efficacy observed with LY2405319 were again nearly identical to observed effects obtained with FGF21 (Figures 8 & 9).

Other attempts to produce engineered human FGF21 variants have recently been reported [28], [33], [34]. Notably, Mu et al. described the introduction of a non-natural amino acid, p-acetylphenylalanine, in the FGF21 sequence for the site-specific attachment of polyethylene glycol (PEG). They characterized activities of their PEGylated FGF21 analogs using both cell-based and animal studies which demonstrated a longer *in vivo* time-action with a pharmacologic profile similar to that of native FGF21. However, these novel PEGylated analogs did not retain the potency of native FGF21, and their potential for undesired mitogenic effects was not examined [33]. A different approach to design an improved FGF21 analog has been communicated by Veniant et al. This report describes the generation of a long-acting FGF21 therapeutic by fusing an immunoglobulin Fc to an FGF21 variant containing two engineered mutations to reduce aggregation and *in vivo* degradation. Expectedly, the resulting FGF21-Fc molecule had an improved half-life when dosed to mice or monkeys, and it appeared to have *in vivo* effects that were generally similar to FGF21 actions in animals [34]. Ye et al. describe a mouse/human FGF21 chimera fused to SUMO protein. The resulting SUMO-hmFGF21 was shown to exert some FGF21-like efficacy *in vitro* and *in vivo* [28]. Taken together with our data, these reports demonstrate that FGF21 can be manipulated in a variety of ways to alter its physico-chemical properties in addition to altering its *in vivo* time action. However, the signaling properties, mitogenic potential, and other aspects of FGF21 pharmacology, as we have describe here for LY2405319, have not been fully characterized with other engineered variants of FGF21 [28], [33], [34]. We have also further addressed the extent to which our FGF21 analog exhibits enhanced stability and may be suitable for larger scale expression-purification and subsequent clinical development. Furthermore, our efforts succeeded in identifying a molecule which closely mimics FGF21 in all respects including *in vivo* potency, exposure and time action. The latter features may be important since there might be undesired consequences of continuous FGF21 action.

In conclusion, the studies reported herein resulted in the identification and characterization of a novel variant of FGF21 which was shown to exhibit markedly improved biophysical features and properties applicable to a multi-use formulation and amenable to large-scale expression. The biologic features of LY2405319 closely matched the profiles observed with FGF21, resulting in a molecule that appears to be sufficiently optimized to study the effects of LY2405319 pharmacologic administration in human patients. Based on extensive studies which employed exogenous native FGF21 in both rodent and non-human primate disease models [1], [19], [21], [24], [29], [33]–[37] and these additional results obtained with LY2405319, there is a compelling rationale to evaluate the potential therapeutic utility of FGF21-based molecules in patients with cardio-metabolic disorders which include metabolic syndrome, obesity, Type 2 diabetes, hepatic steatosis, and atherosclerosis [38].
